# Progress in the application of ultrasound in glioma surgery

**DOI:** 10.3389/fmed.2024.1388728

**Published:** 2024-06-17

**Authors:** Hao Wu, Yingying Cheng, Wenwen Gao, Peng Chen, Yao Wei, Haikang Zhao, Fenglu Wang

**Affiliations:** Department of Neurosurgery, The Second Affiliated Hospital of Xi’an Medical University, Xi’an, China

**Keywords:** intraoperative ultrasound, contrast-enhanced ultrasound, three-dimensional ultrasound, noninvasive ultrasound, ultrasound elastography, glioma, surgery

## Abstract

Brain glioma, which is highly invasive and has a poor prognosis, is the most common primary intracranial tumor. Several studies have verified that the extent of resection is a considerable prognostic factor for achieving the best results in neurosurgical oncology. To obtain gross total resection (GTR), neurosurgery relies heavily on generating continuous, real-time, intraoperative glioma descriptions based on image guidance. Given the limitations of existing devices, it is imperative to develop a real-time image-guided resection technique to offer reliable functional and anatomical information during surgery. At present, the application of intraoperative ultrasound (IOUS) has been indicated to enhance resection rates and maximize brain function preservation. IOUS, which is promising due to its lower cost, minimal operational flow interruptions, and lack of radiation exposure, can enable real-time localization and precise tumor size and form descriptions while assisting in discriminating residual tumors and solving brain tissue shifts. Moreover, the application of new advancements in ultrasound technology, such as contrast-enhanced ultrasound (CEUS), three-dimensional ultrasound (3DUS), noninvasive ultrasound (NUS), and ultrasound elastography (UE), could assist in achieving GTR in glioma surgery. This article reviews the advantages and disadvantages of IOUS in glioma surgery.

## 1 Introduction

Glioma originates from glial cells and is the most common primary intracranial tumor, with an annual incidence of 3–6.4/100,000, accounting for 23.3% of all central nervous system (CNS) tumors and 78.3% of malignant tumors (1). Surgical resection is the main treatment for glioma. However, as a result of the diffuse infiltrating growth of the glioma and the lack of clear boundaries between the glioma and surrounding normal tissue, normal brain tissue and related nerve fiber bundles may be damaged if the glioma is removed only by conventional microsurgery. Therefore, how to accurately locate the tumor, effectively evaluate the tumor boundaries and retain normal nerve function during surgery has become a popular research topic in glioma surgery. Accurate anatomical localization of the nervous system is the basis of neurosurgery, and “precision and precision” is the core tenet of neurosurgery. With the rapid development of intraoperative imaging guidance technology, a variety of medical imaging techniques, including intraoperative neuroelectrophysiological monitoring, intraoperative computed tomography (CT) and magnetic resonance imaging (MRI), fluorescence guidance technology, neural navigation systems, and IOUS, have been gradually applied in neurosurgery to achieve safe glioma resection and to maximize the preservation of patient nerve function. The application of ultrasound in neurosurgery can be traced back to the 1980s. At the beginning, due to issues with imaging quality, its clinical application was relatively limited. However, with the development of science and technology, ultrasound imaging technology has significantly improved, especially owing to the development of 3D ultrasound, color Doppler, contrast ultrasound, linear ultrasound, elastic ultrasound and other methods. The application of IOUS in brain tumor surgery has become increasingly widespread, and neurosurgeons increasingly favor its use, serving as a real guide for the safe resection of tumors and the protection of patients’ neurological function (2).

## 2 Operation sequence

**Figure 1:**
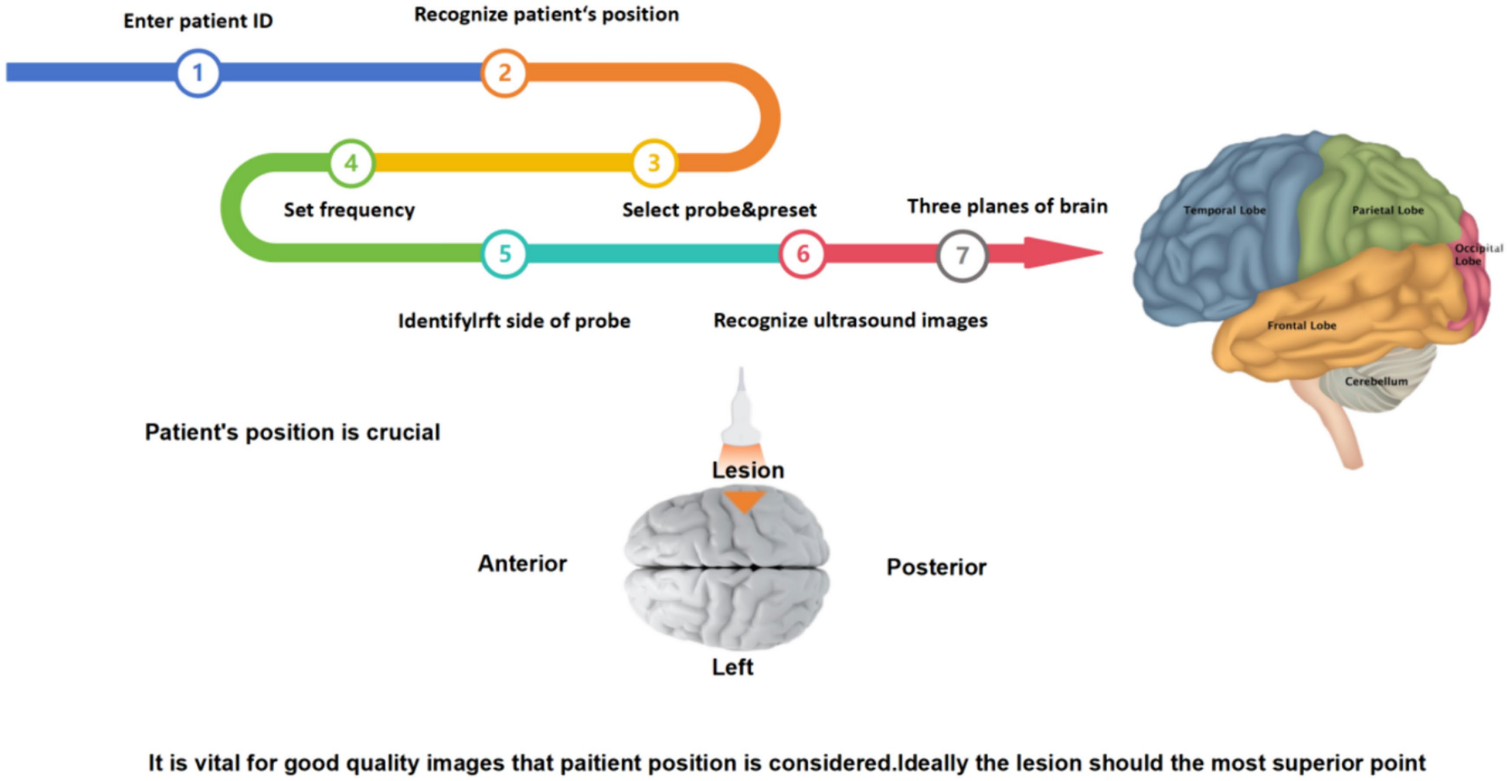
The steps of neurosurgical US scanning.

The steps of intraoperative ultrasound scan in neurosurgery is a specialized and complex medical procedure that involves several key steps to ensure the accuracy and safety of the scan. The brief steps are as follows:

First, before starting a neurosurgical ultrasound scan, adequate preoperative preparation is required, including the patient’s identity, admission number, medical history, examination needs, and assessment of potential complication risk. The doctor also needs to fully communicate with the patient to inform possible risks and complications during the scanning process. Then, depending on the surgical needs, the patient is adjusted in the appropriate position to ensure that the ultrasound scan accurately captures the desired image, and the device is checked to make sure it is in good working order, including check the integrity of the probe, cable, display and other components to ensure image quality. Next, the appropriate ultrasound probe and frequency need to select according to the surgical site. At the same time, the image quality needs to be paid close attention during the scanning process, and the captured ultrasound images are analyzed. Finally, the tumor was located according to different planes (horizontal + coronal + sagittal), and the shape, size, boundary and relationship between the tumor and normal brain tissue were observed to further determine the scope of surgical resection (Figure 1).

**Figure 2:**
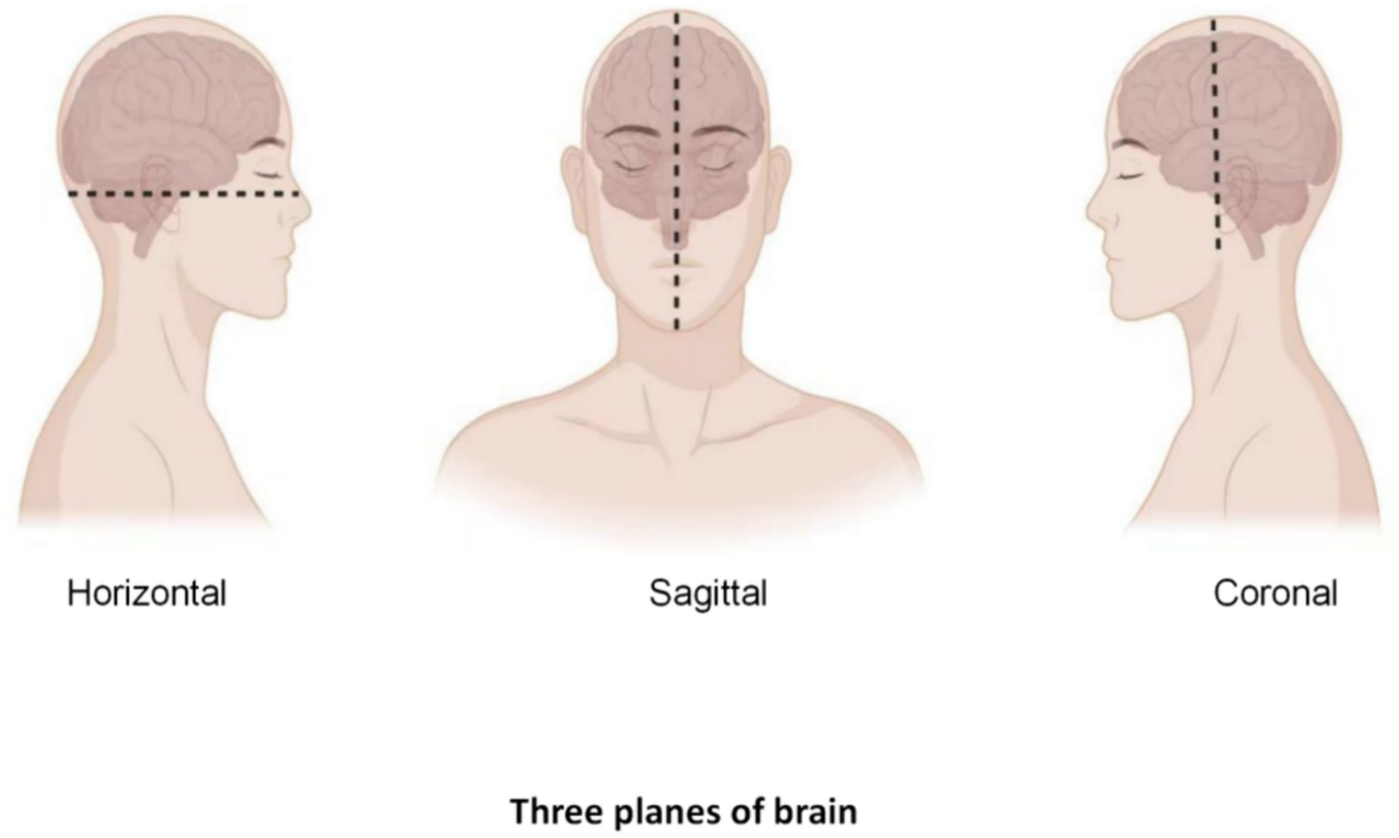
Determination of the three planes.

After routine craniotomy, the dural membrane is cut open, and the probe is placed on the arachnoid surface to glide along with normal saline as a coupling agent. Multiplane detection, such as coronal, sagittal, and horizontal detection, is performed. Two planes (coronal + sagittal or coronal + horizontal) are usually used to visualize the tumor (Figure 2). If the same plane as CT or MRI is obtained, comparative observations can be performed, a clear image can be obtained, the image can be frozen, and the tumor size can be measured and recorded. After finding the lesion, the location of the tumor is determined, and the relationships among the lesion shape, size, boundaries, internal echo (substantive, cystic or mixed), echo homogeneity and normal ventricle structure are observed. The motion of the probe sliding on the arachnoid surface should be gentle and small, and the brain surface should be repeatedly flushed to reduce friction and damage to the brain tissue. After the anatomic boundary of the tumor is determined and labeled by B-ultrasound, the functional boundaries of the tumor (motor, sensory, and language functional areas) are determined, and the tumor is then removed to the maximum extent possible through microscope guidance. After the lesion is resected, the cotton sheet and brain plate are removed from the operative cavity and filled with normal saline. The probe is placed in the operative cavity, and ultrasound scanning is performed to monitor the scope and degree of excision and guide the excision of the residual lesion (3).

## 3 Ultrasonic characteristics of gliomas

Gliomas are often characterized by uneven strong echoes. Due to invasive growth, the boundaries are not clear, and low gyrus vocal cords can be seen around the tumor, which is caused by edema around the tumor. The degree of glioma malignancy is also highly important to ultrasound images. Low-grade gliomas (WHO grade I-II) are usually characterized by uniform internal echoes, regular peritumoral morphology, clear boundaries with the surrounding brain tissue, and no obvious edema. High-grade gliomas (WHO grade III-IV) often present with uneven internal echoes, partial fluid necrosis, irregular peritumoral shapes, unclear boundaries, and obvious edema. Peritumoral edema bands are not obvious in low-grade gliomas, so IOUS has greater significance in differentiating low-grade gliomas (4, 5). The application of IOUS can provide real-time feedback of lesion information and better distinguish between edema and tumor tissue (6).

## 4 Various new developments in IOUS

### 4.1 Contrast enhanced ultrasound (CEUS)

CEUS is a dynamic and continuous imaging method that can observe the vascularization and blood flow distribution patterns of different organs and tumors in real time, improve the resolution, sensitivity and specificity of ultrasound imaging, and evaluate the boundaries for tumor resection (7). CEUS can assist in locating tumors and helping clinicians to accurately and promptly identify the relationships between blood vessels and peripheral vessels (8). CEUS is a significant diagnostic tool that has been widely applied in the liver, lung and heart (9), but its application in glioma surgery is relatively limited (Table 1). Although CEUS can help identify residual tumors during glioma surgery based on changes in local enhancement (16), the application of contrast-enhanced IOUS is limited to evaluating the range of tumor resection in patients with glioma or relapsed glioma after radiotherapy, given that recurrent tumors, radionecrotic tissues and surrounding edema tissues present similar moderate-strong echoes. CEUS allows for real-time angiography, offering valuable information about the parenchyma, as well as the tumor vascularization and perfusion. CEUS can also support glioma detection and surgical planning, which also allows for tumor grade characterization and residue identification. Therefore, the value of CEUS lies in its ability to define the tumor location, evaluate boundaries, distinguish benign and malignant lesions, achieve maximum tumor resection, and enhance the disease-free survival rate of glioma patients (5). Therefore, the use of intraoperative CEUS in neurosurgery will increase step by step.

**Table 1:** Summary of the applications of CEUS for glioma.

Cases	Study points	Conclusion	Time	Ref
87	Due to the infiltrative nature and high local recurrence of gliomas, particularly high-grade gliomas, gross total resection (GTR) of a tumor is the first critical step in treatment. This study aimed to determine whether the integration of intraoperative contrast-enhanced ultrasound (CEUS) and fluorescein sodium can improve the identification of tumor boundaries and residuals, and increasethe extent of resection (EOR) to better protect neurological function.	Intraoperative CEUS with fluorescein sodium is a real-time, straightforward, safe, and effective approach to perform surgical resection of gliomas. This approach assists surgeons in identifying tumor boundaries, residual tumors, and normal brain parenchyma, which increases the EOR.	2024	Fang et al. (10)
64	Complete resection of malignant gliomas is often challenging. Our previous study indicated that intraoperative contrast-enhanced ultrasound (ICEUS) could aid in the detection of residual tumor remnants and the total removal of brain lesions. This study aimed to investigate the survival rates of patients undergoing resection with or without the use of ICEUS and to assess the impact of ICEUS on the prognosis of patients with malignant glioma.	ICEUS facilitates the identification of residual tumors. Age and ICEUS are prognostic factors for malignant glioma surgery, and use of ICEUS offers a better prognosis for patients with malignant glioma.	2024	Chen et al. (11)
51	To investigate the value of routine intraoperative ultrasound (IU) and intraoperative contrast-enhanced ultrasound (ICEUS) in the surgical treatment of brain tumors, and to explore the utilization of ICEUS for the removal of the remnants surrounding the resection cavity.	ICEUS is a useful tool in localizing and outlining brain lesions, especially for the resection of the hypervascular lesions in the brain. ICEUS could be more beneficial for identifying the remnants and improving the rate of total removal of these lesions than routine intraoperative ultrasound.	2022	Tao et al. (12)
49	To analyze the relationship between quantitative CEUS parameters and microvessel density (MVD) in different grades of gliomas	CEUS provides dynamic and continuous real-time imaging and quantitative data analysis of different grades of gliomas; the quantitative CEUS parameters were closely related to MVD and were helpful in understanding glioma grade and optimizing surgical strategy.	2019	Wang et al. (5)
10	To assess the capability of CEUS to identify residual tumor mass during glioma surgery and to increase the extent of resection (EOR)	CEUS is extremely specific in the identification of residual tumor. The ability of CEUS to distinguish between tumor and artifacts or normal brain on B-mode is based on its ability to determine the vascularization degree. Therefore, CEUS can play a decisive role in the process of maximizing GBM resection.	2016	Prada et al. (13)
5	To provide further clinical data on the versatile application of CEUS through a technical note and illustrative case series	CEUS provides safe, real-time, and dynamic contrast-based imaging that can potentially be used for routine neurooncological surgery and image-guided biopsy. CEUS eliminates the effect of anatomical distortions associated with standard neuronavigation and provides quantitative perfusion data in real time, which may hold major implications for intraoperative diagnosis, tissue differentiation, and quantification of EOR.	2016	Lekht et al. (14)
120	To evaluate the diagnostic significance of CEUS in assessing the resection degree of brain glioma using transmission electron microscopic (TEM) examination	CEUS had high sensitivity and specificity for evaluating the extent of tumor excision. Residual tumor rates detected using ultrasound contrast and TEM examination, respectively, had medium consistency. The application of intraoperative contrast-enhanced ultrasound can improve the resection rate of brain glioma	2015	Yu et al. (7)
69	To perform the first characterization of cerebral glioma using CEUS and to possibly achieve intraoperative differentiation of different gliomas	CEUS is a fast, safe, dynamic, real-time, and economic imaging modality that might be helpful in differentiating malignant and benign gliomas during surgery and refining surgical strategies	2014	Prada et al. (15)

### 4.2 Three-dimensional ultrasound (3DUS)

3DUS is based on two-dimensional ultrasound (2DUS) imaging of coronal sections. Since 3DUS is the only field of neurosurgery with such a wide range of applications, it is now widely used in neurosurgery, especially for intraparenchymal tumors (Table 2), cavernomas, skull base tumors, medullary lesions, arteriovenous malformations, and endoscopic guidance. Therefore, 3DUS can be used not only as a diagnostic tool but also to guide surgical strategies. To obtain the best results from 3DUS, neurosurgeons should master more of the principles of using IOUS in glioma surgery.

**Table 2:** Summary of the applications of three-dimensional ultrasound (3DUS) for glioma.

Cases	Study points	Conclusion	Time	Ref
252	Intraoperative ultrasound is a promising tool for intraoperative tumor resection control. Navigated three-dimensional US (n3DUS) has many benefits over standard two-dimensional US (2DUS) boundaries and residuals, and increasethe extent of resection (EOR) to better protect neurological function.	Good delineation, noneloquent location, and use of n3DUS was associated with a higher probability of GTR in glioma surgery. Surgeons’ experience did not influence the extent of resection. Morbidity was predominantly associated with eloquent location, independent of all other factors.	2023	Moiyadi et al. (17)
74	To assess radiological and clinical results in consecutive patients with LGG treated with 3DUS-guided resection under general anesthesia	3DUS-guided LGG resections under general anesthesia are safe and they preserve HRQoL in most patients. Effectiveness in terms of EOR appears to be consistent with published studies using other advanced neurosurgical tools. Avoiding intraoperative vascular injury is a key factor for achieving good functional outcome	2019	Bø et al. (18)
162	To assess 3DUS visibility of different pathologies and IOUS applications during the course of surgery	IOUS was highly sensitive in detecting all types of pathology, was safe and precise in planning trajectories to intraparenchymal lesions (including minimally mini invasive approaches), and was accurate in determining EOR in more than 80% of the cases. IOUS is a safe, versatile, and feasible tool that may be considered for routine intracranial surgery EOR in more than 80% of the cases. IOUS is a safe, versatile, and feasible tool that may be considered for routine intracranial surgery intraoperative ultrasound.	2018	Policicchio et al. (19)
28	To assess the effectiveness of 3DUS during awake resections of eloquent LGGs by comparing surgical results of two series of patients operated on using conventional neuronavigation and 3DUS	The extent of awake resections of eloquent LGG was greaterwith 3DUS guidance than with standard neuronavigation guidance; the use of 3DUS had no impact on the number of new permanent deficits	2017	Šteňo et al. (20)
111	To evaluate the effectiveness of navigable 3DUS as a novel intraoperative imaging adjunct permitting quick real-time updates to facilitate tumor resection	The results of this study demonstrated that 3DUS can be effectively used as a stand-alone navigation modality during the resection of brain tumors. The ability to provide repeated, high-quality intraoperative updates is useful for guiding resection. Attention to image acquisition technique and experience can significantly increase the image quality, thereby improving the overall utility of this modality	2016	Moiyadi & Shetty (21)

### 4.3 Navigable ultrasound (NUS)

NUS is a new technology that can locate and navigate tumors by tracking two-dimensional (2D) or 3DUS images. In addition, this technology is an innovative intraoperative imaging-assisted technology that can be rapidly updated in real time to facilitate tumor removal (Table 3).

**Table 3:** Summary of the applications of navigable ultrasound (NUS) for glioma.

Cases	Study points	Conclusion	Time	Ref
210	Intraoperative imaging is increasingly being used for resection control in diffuse gliomas, in which the extent of resection (EOR) is important. Intraoperative ultrasound (iUS) has emerged as a highly effective tool in this context. Navigated ultrasound (NUS) combines the benefits of real-time imaging with the benefits of navigation guidance. In this study, the authors investigated the use of NUS as an intraoperative adjunct for resection control in gliomas.	NUS is a useful intraoperative adjunct for resection control in gliomas, detecting unanticipated tumor residues and positively influencing the course of the resection, eventually leading to higher resection rates. Nevertheless, resection is determined by the innate resectability of the tumor and its relationship to eloquent location, reinforcing the need to combine iUS with functional mapping techniques to optimize resections.	2021	Shetty et al. (22)
31	To evaluate the impact of real-time conventional neuronavigation combining NUS and preoperative magnetic resonance imaging (MRI) on maximizing EOR in glioma surgery compared to standard conventional neuronavigation	The use of NUS-based real-time imaging modality promoted better EOR and neurological outcomes following the resection of noneloquent high-grade gliomas compared to standard conventional neuronavigation. NUS has proven to be useful in detecting RTV > 1 cm^3^	2020	Moiraghi et al. (23)
125	To evaluate the relative utility and benefits of free-hand 2DUS and navigated 3DUS as ultrasound-guided biopsy techniques for supratentorial lesions	Despite the longer operative time and higher postoperative complication rates, NUS was beneficial for biopsies of deep- seated supratentorial lesions, while free-hand 2DUS remained valuable for superficial lesions EOR in more than 80% of the cases. IOUS is a safe, versatile, and feasible tool that may be considered for routine intracranial surgery EOR in more than 80% of the cases. IOUS is a safe, versatile, and feasible tool that may be considered for routine intracranial surgery intraoperative ultrasound.	2019	Patil et al. (24)
11	To assess whether the combined use of navigated ultrasonography integrating FMRIB software library-based probabilistic fiber tracking into neuronavigation was technically feasible and achievable in the preoperative and intraoperative workflow	Integration of probabilistic fiber tracking and navigated ultrasonography into intraoperative neuronavigation facilitated anatomic orientation during glioma resection. Combination with NUS provided a three-dimensional estimation of intraoperative brain shift, thereby improving the reliability of neuronavigation	2016	Rueckriegel et al. (25)

### 4.4 Ultrasound elastography

Ultrasound was first introduced into clinical practice in the 1970s (26). Since then, new ultrasound techniques, such as Doppler imaging, have been developed, providing new information for diagnosis and real-time control of tumor resection (27). Elastography, the science of creating noninvasive images of the mechanical features of tissue, has developed rapidly in recent years (Table 4).

**Table 4:** Summary of the applications of ultrasound elastography (UE) for glioma.

Cases	Study points	Conclusion	Time	Ref
36	To determine the elastographic patterns of different brain tumor types and establish differences between their peritumoral regions.	We objectively described the elastographic patterns of different types of brain tumors. We identified differences in both the tumors and peritumoral areas according to histologic types.	2020	Cepeda et al. (28)
64	To describe the first large-scale implementation of strain elastography (SE) in oncological neurosurgery for lesions discrimination and characterization.	SE allows clinicians to understand the mechanical properties of the brain and lesions during examination and permits better discrimination between different tissues compared to B-mode. Additionally, SE can differentiate between LGG and HGG.	2019	Prada et al. (29)
63	To characterize elasticity of the normal brain parenchyma and brain tumors using shear-wave elastography (SWE) for supratentorial lesions.	Significant differences in elasticity were observed among the most common types of brain tumors. With intraoperative SWE, neurosurgeons may acquire innovative information to establish a diagnosis and guide resection.	2016	Chauvet et al. (30)

## 5 Application of intraoperative ultrasound in glioma surgery

The main objective of brain tumor surgery is to remove as much tumor tissue as possible and reduce recurrence rates. Intraoperative navigation has become the standard for preresection initial localization and evaluation of tumor boundaries in many hospitals. Multiparametric IOUS is attractive due to its abovementioned advantages (31) and has been used mainly for intraoperative localization, evaluation of the resection range, and real-time monitoring of residual tumors.

### 5.1 Intraoperative localization

**Figure 3:**
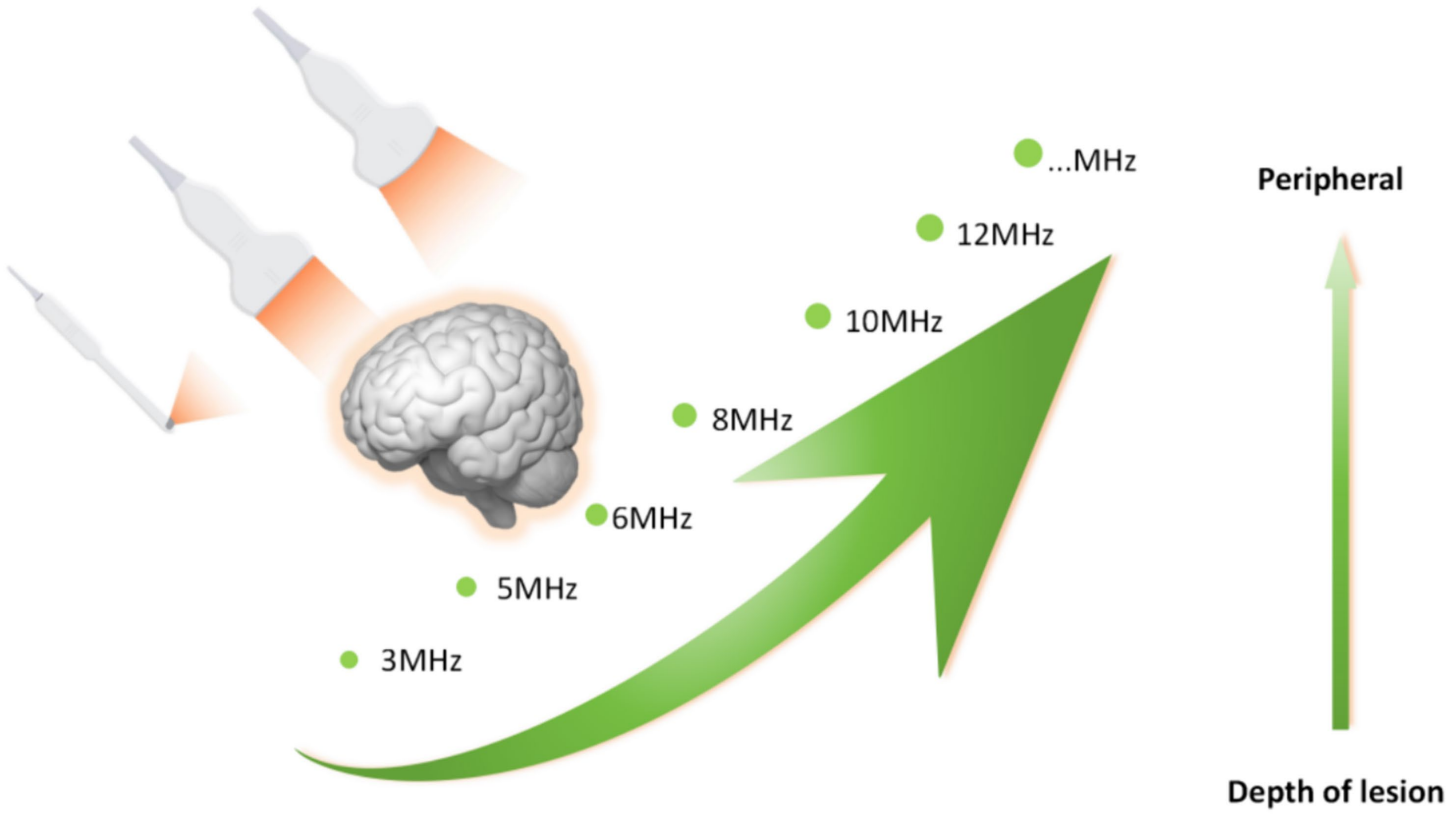
Different probes for real-time imaging and corresponding field depth.

Glioma can occur in various parts of the brain, and due to its extensive invasion, unclear boundaries and other characteristics, preoperative surgical planning is the first step in determining whether an operation will be successful. Most neurosurgical tumor centers rely on preoperative craniocerebral CT and MRI combined with neuronavigation to design specific surgical plans, including optimal preoperative surgical incisions and dynamic localization of the tumor sites after craniotomy. However, when the surgeon incises the dural membrane, due to the influence of factors such as changes in the patient’s surgical position, head frame displacement, gravity, slow release of cerebrospinal fluid, and brain edema, the actual anatomical location and tumor location are inconsistent with the preoperative imaging data, which seriously affects the accuracy of tumor resection, resulting in incomplete surgery and an increased risk of neurological dysfunction. According to Stieglitz’s study, the average displacement of the dura after removing the bone flap was approximately 1.2 mm, and that of the cerebral cortex after cutting the dura was approximately 4.4 mm (32). It has also been reported that displacement of the cerebral cortex can occur in the range of 4.4–20.0 mm during craniotomy. To correct the deviation caused by brain displacement, clinical researchers use intraoperative MRI to accurately reflect the specific location of the tumor and the boundary of the normal brain tissue and to guide the surgeon to accurately remove the tumor tissue. However, intraoperative MRI also has obvious shortcomings. First, it is expensive and brings additional economic burden to patients. Second, MRI equipment occupies a large space. Third, the use of intraoperative MRI navigation undoubtedly extends the operation time and potentially increases the risk of infection. Finally, real-time monitoring of tumors by intraoperative navigation via MRI cannot provide continuous tumor imaging, which significantly limits its application in neurosurgery. IOUS scanning of tumors can overcome the shortcomings of the above imaging methods. In terms of correcting the problem of “brain displacement,” IOUS can also monitor the specific location of the tumor (including the depth and span of tumor infiltration) and the range of normal brain tissue around the tumor in real time. Not only can the tumor lesions be removed to the maximum extent possible but also the collateral damage can be reduced to the minimum (33, 34). In practice, most researchers have clarified and defined the role of ultrasound in surgery: before opening the dural membrane, it can detect lesions, determine the extent of the lesions, and adjust the surgical approach accordingly if necessary. Once the dural membrane is opened, although brain displacement may occur and the anatomical structure may change, IOUS can still monitor lesions, adjacent anatomical marks and important structures (35). In addition, ultrasound can also select different probes for real-time navigation and positioning according to the distance between the tumor and the cortex. Linear or multifrequency [(3–11) MHz] probes are used for deep lesions, and high-frequency [(10–22) MHz] probes are used for superficial lesions (Figure 3). The use of different probes makes the surgical plan more individualized to achieve maximum effective resection. Better image quality is more conducive to real-time intraoperative navigation. 3D IOUS has more advantages than 2D ultrasound in terms of image quality, navigation and subsequent image acquisition. Through research, some scholars have found that 3D ultrasound can safely guide the resection of low-grade gliomas in real time and the results are comparable to those of intraoperative MRI. With the advancement of ultrasound imaging technology, IOUS can be used to accurately locate tumors, minimize damage, improve surgeons’ efficiency in glioma surgery, enhance confidence, and guide the selection of the surgical treatment for glioma (36).

### 5.2 Determining the scope of tumor resection

The surgical principle of gliomas is to safely remove the tumor in the largest extent possible to avoid damage to the normal brain tissue, and it is highly important to determine the tumor resection boundary and monitor the surgical resection scope (37, 38). High-grade gliomas usually have unclear boundaries due to their high degree of malignancy and obvious peritumoral edema, making it difficult to distinguish normal boundaries, while low-grade gliomas are often permeated, infiltrated and similar in appearance to normal brain tissue, making them difficult to distinguish from normal brain tissue. Trevisi’s study showed that the comprehensive sensitivity and specificity of IOUS for evaluating the scope of tumor resection were 72.2 and 93.5%, respectively. Due to the high sensitivity of ultrasound, gliomas can be well identified in ultrasound images, and the imaging boundaries of tumors can be clearly displayed. The echo of the glioma on the ultrasound image depends on the cell density. The intrinsic component of high-grade gliomas is a typical medium-high echo. At the same time, high-grade gliomas echo unevenly, with different necrotic areas (medium-low echo), cysts (very low echo), bleeding areas (different degrees of echo depending on the time) and peritumoral edema (high echo), while low-grade gliomas echo slightly less than high-grade gliomas, and the echo is uniform, resulting in clearer boundaries (39). Hou et al. (40) observed in their prospective study that IOUS, compared with intraoperative MRI before and during tumor resection, can more accurately display the resection boundaries and control the resection process according to these boundaries by utilizing the difference in echo between the tumor and normal tissue. Similar to MRI, it can be difficult to distinguish between invasive tumors and peripheral edema with ultrasound because both can be hyperechoic. To distinguish the boundary between the two, CEUS technology has been gradually applied in the clinic. CEUS is an ultrasound examination method that uses contrast agents and specific algorithms to study the cerebrovascular system. By providing dynamic imaging and functional or perfusion data, CEUS can visualize the degree of vascularization of the tumors and normal tissues and further identify the tumor boundaries (41). Wang et al. found that the intraoperative CEUS features of normal brain tissue were “grid-like” with moderate enhancement, and the edema area around the lesion showed low contrast enhancement, while the enhancement degree of high-grade glioma was significantly increased, which helped distinguish between edema and tumor tissue. Therefore, ultrasound can provide real-time echo morphology of different pathological tissues and better distinguish the boundaries between gliomas at all levels and normal tissues (5).

### 5.3 Real-time monitoring of residual tumors

As more research has been done on gliomas, studies have shown that a smaller tumor residue after surgery is associated with improved survival. Accurate removal of gliomas and a reduction of tumor residues are difficult for surgeons in most cases. Although the current intraoperative image-guided technology can improve the resection rate of residual tumors, compared with IOUS, it has greater accuracy in real-time and continuous imaging and the detection of glioma residues. Other methods, such as intraoperative CT and MRI, are difficult to widely promote in clinical practice. Zhang et al. found through meta-analysis that both the sensitivity and area under the curve were greater for low-grade gliomas than for high-grade gliomas. The Deeks plot showed no significant publication bias (t = −1.03, *p* = 0.33). IOUS has high diagnostic significance for identifying glioma remnants, especially in low-grade gliomas, which indicates a benefit for patient prognosis and quality of life. In summary, IOUS is an available tool for maximizing the extent of glioma resection (42). IOUS has a sensitivity of 75% and specificity of 88% for glioma residues, especially for low-grade gliomas, with a sensitivity of up to 87%, which can help surgeons make more informed decisions interoperatively and thus improve tumor removal rates. During surgery, due to the influence of tissue pulling, tumor resection, bleeding and hemostatic materials, the image quality and diagnostic sensitivity and specificity of ultrasound gradually decrease, thus affecting the degree of tumor resection. Munkvold et al. demonstrated in a prospective study of 144 glioma patients that the overall sensitivity of “no tumor residues” in ultrasound images at the end of resection was 46%, and the specificity was 85%. In view of this defect, clinical researchers have made active innovations, and the emergence of ultrasound in linear array surgery has effectively improved this shortcoming (43). The overall specificity of “no tumor residues” in IOUS images at the end of resection is 85% compared to postoperative MRI results. The sensitivity is 46%, but in surgeries where there is a false-negative on ultrasound, the residual tumor seen on MRI is usually small (median 1.05 mL). The specificity was highest in patients who underwent low-grade glioma surgery (94%) and lowest in patients who received radiation therapy (50%). According to the multivariate logistic regression analysis, small tumor volume and body surface area were factors associated with total resection, while satisfactory ultrasound image quality was not statistically significant (*p* = 0.061). Their results showed that the specificity of IOUS was better, but the sensitivity for detecting the last milliliter was relatively low compared with that of postoperative MRI. Tumor volume and tumor depth are predictive indices of gross total resection, although ultrasound image quality is not. In glioma surgery, we analyzed the sensitivity, specificity, and predictive value of 3D-IOUS in detecting residual tumors compared to early postoperative MRI. Linear ultrasound provides higher-quality images, especially in regard to visualizing tumor residues. During surgery, linear ultrasound did not show the same degradation in image quality as conventional ultrasound, and it was used more frequently in the later stages of surgery than conventional ultrasound probes. Studies have shown that the ability of linear ultrasound to detect residual tumors in high-grade gliomas is similar to that of intraoperative MRI, with higher sensitivity and similar specificity. It is an important way to detect small tumors and tumor infiltration and can sometimes directly distinguish normal tissue from pathological tissue (23).

## 6 Limitations of IOUS

Compared with other intraoperative guidance techniques, ultrasound provides a simpler, more economical, more flexible, and more effective intraoperative guidance technique, but it also has inherent defects: (1) strong professionalism, resulting in a long learning cycle, and the lack of specific training for many neurosurgeons, which makes it difficult to identify anatomical structures via preoperative CT or MR imaging; (2) the quality of the image often depends on the skill of the operator and due to variability in the experience of the operator, the image quality is often uneven; and (3) the skull cannot be penetrated before surgery, the imaging quality is not as good as that of CT and MRI, and the resolution of deep brain lesions is low, especially for skull base lesions. However, emerging ultrasonic probes, couplings and various modes of ultrasound technology can, to a certain extent, reduce the impact of the above defects, particularly 3D ultrasound, which provides a more stereoscopic display of the intracranial anatomy and further improves image quality. Previous studies have shown that the resection rate of high-grade gliomas with 3D ultrasound systems can reach 92%, and for low-grade glioma patients with functional areas undergoing awake surgery, the resection rate of patients with 3D ultrasound navigation is greater (20, 31). The application of various probes and new couplings has greatly improved image resolution. The use of novel couplings has been proven to reduce surgically-induced ultrasound artifacts by inserting smaller ultrasound probes into the operative cavity to observe suspicious areas at close range, thereby reducing the impact of artifacts (44, 45).

## 7 Discussion

The surgical management of eloquent area gliomas is a new challenge for neurosurgeons. In recent years, with the application and development of intraoperative ultrasound, intraoperative cortical electrical stimulation and other technologies, lesions and functional fields can be accurately located during surgery, which greatly enhances the surgical therapeutic effect on functional gliomas, as well as the postoperative survival time and quality of life of patients (46).

In traditional glioma surgery, lesion localization primarily depends upon the neurosurgeon’s understanding and experience with imaging data. After craniotomy, the brain sulcus depth, gyrus shape, color and blood vessel flow are observed and estimated (1, 47, 48). The above indications cannot be used to accurately evaluate the entire lesion contour. In recent years, however, neural navigation systems have helped to distinguish the location of tumors during surgery. The reference frame of the navigation system is preoperative MRI or CT, but intraoperative shifts in brain tissue will ineluctably impact the precision of neural navigation systems (49, 50). Due to effects from the shape of the bone window and intracranial pressure (ICP), it is difficult to predict the direction of lesion tissue shift. Because IOUS can resolve the issue of intraoperative brain tissue shift and is easy to perform, provides clear images, and is inexpensive, its real-time localization role in neurosurgery has attracted increasing attention. Several studies have verified its good application value in determining the range of tumors, enhancing the entire resection rate of tumors, and decreasing collateral damage.

Currently, ultrasound has the following advantages: small specific volume; clear, high-definition images; convenient and easy intraoperative application; shortened operation time; and the avoidance of additional injuries. During the resection of gliomas in the brain functional area, IOUS is applied to fix the tumor and determine the extent of tumor resection in real time. If IOUS can be combined with cortical electrical stimulation to locate the functional area, it will be more useful to judge the relationship between the tumor and the functional area, which is very valuable for transcortical surgery. For nonfunctional tumors, corticostomy should be performed as close to the lesion as possible and with the smallest incision. For eloquent area tumors, ultrasound can be used to remove the tumor while protecting the cortex of significant brain functional fields to the greatest extent possible, decreasing the incidence of postoperative limb dysfunction. After tumor resection, ultrasound examination can be conducted again to assess the extent of lesion resection and determine whether there are tumor residues in the brain via a microscope, which can increase the total resection rate. However, ultrasound images are influenced by several factors, spatial defects still exist, and cross-sectional images differ from those of the normal anatomy. We firmly believe that with the development of smaller and more accurate probes, increased transparency of ultrasonic images, enhanced understanding of ultrasonic pictures, the emergence of 3DUS, and the use of a combination of ultrasound and microneurosurgery technology, neuroendoscopy technology, lasers, neural navigation and other progressive technologies, the application of intraoperative real-time ultrasonic localization in functional area glioma surgery will improve.

## 8 Conclusion

Maximal secure resection of brain tumors is a key treatment in glioma surgery. In most instances, the intraoperative range of the resection can be accurately estimated by IOUS. With further technical improvements and the application of other advanced visualization tools during surgery, we firmly believe that intraoperative tumor removal will be more accurate.

## 9 Summary and prospects

IOUS has become an important intraoperative auxiliary technology in the field of neurosurgery. The advantages of each imaging mode (B-ultrasound, linear ultrasound, contrast ultrasound, elastography) include its potential for accurately locating lesions, determining boundaries, identifying residual tumors, and guiding glioma resection. The degree of glioma resection is the most important factor in determining the survival time and quality of life of patients. Due to the aggressive growth of gliomas, it is difficult to accurately locate the boundaries of gliomas by looking at their color and texture with the naked eye or microscope. IOUS has become an important tool in neurosurgery, and it has high sensitivity and specificity in judging the degree of resection of brain gliomas. In addition to reducing the artifacts of brain tumors and predicting histological diagnosis, advances in ultrasound technology have improved the resolution and quality of ultrasound images. There is an urgent need for advances in ultrasound imaging processing, as well as continuous improvements in hardware, to fully realize the potential of IOUS. Therefore, the application of IOUS in intracranial glioma surgery can improve the resection rate of tumors, reduce tumor residues, and improve the survival rate and quality of life of patients, and is an important auxiliary technology in brain glioma surgery.

## Author contributions

HW: Writing – original draft, Writing – review & editing. YC: Writing – original draft, Writing – review & editing. WG: Conceptualization, Software, Writing – review & editing. PC: Writing – original draft. YW: Formal analysis, Investigation, Software, Writing – original draft. HZ: Project administration, Resources, Validation, Visualization, Writing – review & editing. FW: Data curation, Writing – review & editing.
